# Risk factors for nephrotoxicity after ifosfamide treatment in children: a UKCCSG Late Effects Group study

**DOI:** 10.1054/bjoc.2000.1214

**Published:** 2000-04-27

**Authors:** R Skinner, S J Cotterill, M C G Stevens, on behalf of the Late Effects Group of the United Kingdom Children’s Cancer Study Group (UKCCSG)

**Affiliations:** Sir James Spence Institute of Child Health, Royal Victoria Infirmary, Queen Victoria Road, Newcastle upon Tyne, NE1 4LP, UK; Birmingham Children's Hospital, Steelhouse Lane, Birmingham, B4 6NH, UK

**Keywords:** ifosfamide, nephrotoxicity, children, adolescents, cancer

## Abstract

The aim of this multicentre study was to document the nephrotoxicity associated with ifosfamide and evaluate risk factors in 148 children and young people with sarcomas who underwent investigation of renal function on one occasion each, at a median of 6 (range 1–47) months after completion of ifosfamide (median dose 62.0 (range 6.1–165.0) g/m^2^). Investigations included glomerular filtration rate (GFR), serum bicarbonate (HCO_3_) and phosphate (PO_4_), and renal tubular threshold for phosphate (Tm_p_/GFR). A clinically relevant ‘nephrotoxicity score’ was derived. GFR was < 90 ml/min/1.73 m^2^in 61 of 123 evaluable patients, Tm_p_/GFR < 0.9–1.1 mmol/l (age-dependent) in 45/103, serum PO_4_< 0.9–1.mmol/l (age-dependent) in 28/135, and serum HCO_3_< 20 (< 18 in infants) mmol/l in 22/95. Of 76 fully evaluable patients: 50% had mild, 20% moderate and 8% severe nephrotoxicity. Higher total ifosfamide dose correlated significantly with greater glomerular and tubular toxicity (*P*< 0.01); other risk factors, including age at treatment, demonstrated no consistent significant independent effect. Chronic ifosfamide-related glomerular and proximal tubular toxicity were common in this large comprehensive study. Restriction of total ifosfamide dose to < 84 g/m^2^will reduce the frequency of, but not abolish, clinically significant nephrotoxicity, whilst doses > 119 g/m^2^are associated with a very high risk of severe toxicity. © 2000 Cancer Research Campaign


*British Journal of Cancer* (2000) **82**(10), 1636–1645

© 2000 Cancer Research Campaign

DOI: 10.1054/ bjoc.2000.1214, available online at http://www.idealibrary.com on IDEAL®

### Risk factors for nephrotoxicity after ifosfamide treatment in children: a UKCCSG Late Effects Group study

**R Skinner[1], SJ Cotterill[1] and MCG Stevens[2] on behalf of the Late Effects Group of the United Kingdom Children's Cancer Study Group (UKCCSG)**

[1]Sir James Spence Institute of Child Health, Royal Victoria Infirmary, Queen Victoria Road, Newcastle upon Tyne NE1 4LP, UK; [2]Birmingham Children's Hospital, Steelhouse Lane, Birmingham B4 6NH, UK

**Summary** The aim of this multicentre study was to document the nephrotoxicity associated with ifosfamide and evaluate risk factors in 148 children and young people with sarcomas who underwent investigation of renal function on one occasion each, at a median of 6 (range 1–47) months after completion of ifosfamide (median dose 62.0 (range 6.1–165.0) g/m$^2$). Investigations included glomerular filtration rate (GFR), serum bicarbonate ($HCO_3$) and phosphate ($PO_4$), and renal tubular threshold for phosphate ($Tm_p$/GFR). A clinically relevant 'nephrotoxicity score' was derived. GFR was < 90 ml/min/1.73 m$^2$ in 61 of 123 evaluable patients, $Tm_p$/GFR < 0.9–1.1 mmol/l (age-dependent) in 45/103, serum $PO_4$ < 0.9–1.mmol/l (age-dependent) in 28/135, and serum $HCO_3$ < 20 (< 18 in infants) mmol/l in 22/95. Of 76 fully evaluable patients: 50% had mild, 20% moderate and 8% severe nephrotoxicity. Higher total ifosfamide dose correlated significantly with greater glomerular and tubular toxicity ($P < 0.01$); other risk factors, including age at treatment, demonstrated no consistent significant independent effect. Chronic ifosfamide-related glomerular and proximal tubular toxicity were common in this large comprehensive study. Restriction of total ifosfamide dose to < 84 g/m$^2$ will reduce the frequency of, but not abolish, clinically significant nephrotoxicity, whilst doses > 119 g/m$^2$ are associated with a very high risk of severe toxicity. © 2000 Cancer Research Campaign

**Keywords**: ifosfamide; nephrotoxicity; children; adolescents; cancer

The great improvements in treatment for malignant disease in childhood have led to a major increase in the number of long-term survivors, reaching approximately 1 in every 900 young adults in the USA by the year 2000 (Bleyer, 1990). Much of the improvement in prognosis over the last 30 years has been due to the use of effective multiagent chemotherapy. However, the late adverse effects of this treatment may impair normal development and maturation in children, cause lifelong ill health or disability, or even lead to premature death (Hawkins and Stevens, 1996). The rational development of preventive strategies depends on detailed documentation and analysis of such toxicity.

Ifosfamide is an alkylating oxazaphosphorine with considerable activity against a wide range of malignancies in both adults and children (Zalupski and Baker, 1988; Pratt et al, 1989). It is used increasingly in children since it may have advantages over cyclophosphamide, its structural isomer, especially in the treatment of Ewing's sarcoma and rhabdomyosarcoma (Zalupski and Baker, 1988; Pratt et al, 1989). However, the relative merits of ifosfamide and cyclophosphamide have been the subject of considerable debate (Shaw and Eden, 1990). Moreover, ifosfamide may cause a characteristic pattern of nephrotoxicity in up to 30% of children (Skinner et al, 1993). Once established, such damage is persistent in most patients and may limit the ability to deliver optimum potentially curative chemotherapy subsequently.

*Received 2 August 1999*
*Revised 27 December 1999*
*Accepted 24 January 2000*

*Correspondence to:* R Skinner

There is little information concerning the frequency of long-term toxicity in children due to the paucity of follow-up studies. Most well documented cases of severe nephrotoxicity have occurred in children younger than 5 years old or in children receiving higher cumulative ifosfamide doses (Skinner et al, 1993). A recent study reported that prior cisplatin treatment or nephrectomy increased the risk of nephrotoxicity (Rossi et al, 1994). However, the relative importance of these and other patient- and treatment-related risk factors for the development of renal damage remains unclear.

In view of this data, the Late Effects Group of the United Kingdom Children's Cancer Study Group (UKCCSG) performed a large and comprehensive study of renal function in children and adolescents previously treated with ifosfamide in UKCCSG centres. The aims were to investigate the prevalence, nature and severity of chronic nephrotoxicity and the relevance of patient- and treatment-related risk factors.

### METHODS

### Patients

Any child, adolescent or young adult, who had completed treatment that included ifosfamide at a UKCCSG centre was eligible for this cross-sectional study. Most patients had participated in the UKCCSG ET-2 trial (Craft et al, 1998) (Ewing's sarcoma [ES] of bone), which opened to recruitment in 1987, or in the International Society of Paediatric Oncology (SIOP) MMT-89 trial (rhabdomyosarcoma [RMS], soft tissue sarcoma [STS], extraosseous ES, or primitive neuroectodermal tumour [PNET]), which opened in 1989. Examination of the ET-2 and MMT-89 databases in 1991 revealed 119 surviving patients who had completed treatment with ifosfamide-containing schedules at the 11 participating centres. Of these, 91 (77%) were studied, but 17 (14%) were no longer available for study due to death or disease progression. Only 11 (9%) potentially available patients were not studied. An additional 57 patients treated with ifosfamide using protocols other than ET-2 or MMT-89 were studied at four of the participating centres. Twenty of these patients have been included in a previous single centre report (Skinner et al, 1996). In total, therefore, 148 patients were studied. All but one received ifosfamide at their initial presentation, the other patient at first relapse.

Between 1992 and 1994 each patient was studied once prospectively at a median of 6 (range 1–47) months after completion of chemotherapy. Their median age at commencement of ifosfamide treatment was 8.1 (range 0.1–25.2) years; 87 were male. Only one was over 20 years of age.

The patients had been treated for RMS (65 patients), ES of bone (59), other STS (16), extraosseous ES or PNET (seven), or osteosarcoma (one). At commencement of ifosfamide treatment, one patient with lower urinary tract obstruction had a raised serum creatinine concentration; no other patient had clinical evidence of abnormal renal function (raised serum creatinine and/or glomerular filtration rate [GFR] < 60 ml/min/1.73 $m^2$). Although no patient had undergone a nephrectomy, and none had renal infiltration by tumour, nine (including the child described above) had urinary tract obstruction due to tumour at initial diagnosis of their malignancy.

### Treatment

Ifosfamide was given intravenously (i.v.) by continuous infusion (6–9 g/$m^2$ $course^{-1}$ over 48–72 h) in 93 patients (including 12 in whom a minority of courses were given by short (1–3 h) infusions); by short (3 h) infusions (3 g/$m^2$) on 3 successive days per course (i.e. 9 g/$m^3$ $course^{-1}$) in 53 (including two in whom a minority of courses were given by continuous infusion); or by both schedules (each being used for an equal number of courses) in two.

The median total ifosfamide dose was 62.0 (6.1–165.0) g/$m^2$, given over a median of 8 (1–18) courses at 3-week intervals. All patients received continuous i.v. hydration fluid as specified by the treatment protocols, and all received a continuous i.v. infusion of mesna, with additional boluses in nine. Radiotherapy was given to a treatment volume that included renal tissue in four patients, whilst other potentially nephrotoxic chemotherapy or i.v. supportive treatment was given to 121 patients; three received cisplatin, 107 aminoglycoside antibiotics, 44 vancomycin, 38 acyclovir and 24 standard amphotericin B.

### Investigation and grading of nephrotoxicity

The study protocol was approved by the Joint Ethics Committee of Newcastle Health Authority and the University of Newcastle upon Tyne, and allowed assessment of glomerular, proximal and distal renal tubular function (Skinner et al, 1991).

### *Glomerular function*

GFR and serum creatinine concentration were measured. The plasma clearance of $^{51}$chromium-labelled ethylenediaminetetraacetic acid ($^{51}$Cr-EDTA) was recommended as the method of choice to determine GFR, and was used in 116 patients. GFR was measured from the plasma clearance of $^{99m}$technetium-labelled diethylenetriaminepentaacetic acid ($^{99m}$Tc-DTPA) in a further seven patients.

### *Proximal tubular function*

Concentrations of electrolytes, creatinine, calcium, magnesium, $PO_4$ and glucose were measured in corresponding serum and urine samples. The $HCO_3$ was also measured. The fractional excretions of sodium, potassium, calcium, magnesium, phosphate (FEp), glucose (FEg) and urate, and the renal tubular threshold for phosphate ($Tm_p$/GFR) were calculated using standard formulae (Skinner et al, 1991). The fractional excretion of a substance is the percentage of the filtered load at the glomerulus that is subsequently excreted in the urine; an abnormally high fractional excretion indicates reduced tubular reabsorption. Except for FEg, a fractional excretion was considered abnormal only when elevated in the presence of a reduced serum concentration. The $Tm_p$/GFR provides a measure of tubular phosphate reabsorption, being reduced in the presence of impaired reabsorption.

### *Distal tubular function*

The early morning urine pH and osmolality (EMUO) were measured in the first sample voided on the day of study. Achievement of a pH of ≤ 5.4 was taken to demonstrate normal urinary acidification, and an EMUO of ≥ 600 mOsm/kg to indicate adequate urinary concentration. However, failure to reach these values in a single early morning urine sample was not considered proof of *abnormal* distal tubular function.

### *General aspects of renal function*

The urinary concentrations of albumin and total protein were measured, and expressed as ratios to the simultaneous urine creatinine concentration. Systolic blood pressure was also measured.

### *Nephrotoxicity grading*

This was performed using a previously described system (Skinner et al, 1993), which comprises measurement and scoring of GFR, $Tm_p$/GFR, serum $HCO_3$ and EMUO. These measures were selected to give an overall measure of *clinically important* nephrotoxicity due to ifosfamide, reflecting those aspects of toxicity with the potential to cause morbidity or require chronic treatment. Each was scored on a 0–4 scale, with 0 representing no, 1 mild, 2–3 moderate and 4 severe toxicity within each individual aspect of renal damage. The individual scores are summated to give a ‘nephrotoxicity score’, potentially ranging from 0 to 16 (Table 1). Other individual measures of nephrotoxicity were also graded on a 0–4 scale, and categorized as showing either no/mild toxicity (grades 0 and 1; defined as serum sodium concentration ≥ 121 mmol/l in children < 12 months age or ≥ 126 mmol/l for ≥ 1 year age, serum potassium ≥ 3.0 mmol/l, serum calcium ≥ 1.95 mmol/l, and serum magnesium ≥ 0.60 mmol/l for < 2 years age or ≥ 0.55 mmol/l for ≥ 2 years age), or moderate/ severe toxicity (grades 2–4; defined as serum sodium, potassium, calcium or magnesium concentrations lower than those listed above for grades 0 and 1).

### Normal ranges

The normal ranges for serum biochemistry and fractional excretions were derived from investigation of 105 otherwise healthy

© 2000 Cancer Research Campaign 

**Table 1** Grading criteria for ifosfamide nephrotoxicity

| Nephrotoxicity grade | GFR | $Tm_p$/GFR | | $HCO_3$ | | EMUO |
|---|---|---|---|---|---|---|
| | | < 12 months | ≥ 1 year | < 12 months | ≥ 1 year | |
| 0 | ≥ 90 | ≥ 1.10 | ≥ 1.00 | ≥ 18.0 | ≥ 20.0 | ≥ 600 *or* normal response to DDAVP (if tested) |
| 1 | 60–89 | 0.90–1.09 | 0.80–0.99 | 15.0–17.9 | 17.0–19.9 | 500–599 |
| 2 | 40–59 | 0.70–0.89 | 0.60–0.79 | 12.0–14.9 | 14.0–16.9 | 400–499 |
| 3 | 20–39 | No symptoms, but 0.60–0.69 | 0.50–0.59 | No symptoms, but 10.0–11.9 | 12.0–13.9 | No symptoms, but 300–399 with no response to DDAVP (if tested) |
| 4 | ≤ 19 | HR or myopathy or ≤ 0.60 | ≤ 0.50 | HCMA or ≤ 10.0 | ≤ 12.0 | NDI or ≤ 300 with no response to DDAVP (if tested) |

A score of 4 in an individual aspect of grading (e.g. GFR) constitutes severe toxicity in that aspect. Nephrotoxicity score ($N_s$) = sum of GFR + $Tm_p$/GFR + $HCO_3$ + EMUO. 0 No nephrotoxicity, 1–3 Mild nephrotoxicity, 4–7 Moderate nephrotoxicity, ≥ 8 Severe nephrotoxicity. GFR = glomerular filtration rate (ml/min/1.73 $m^2$) (i.e. evaluating glomerular dysfunction); $Tm_p$/GFR = renal tubular threshold for phosphate (mmol/l) (i.e. evaluating phosphaturia); $HCO_3$ = blood bicarbonate concentration (mmol/l) (i.e. evaluating acidosis); EMUO = early morning urine osmolality (mOsm/kg) (i.e. evaluating impairment of urine concentration); HR = hypophosphataemic rickets, HCMA = hyperchloraemic metabolic acidosis (i.e. renal tubular acidosis), NDI = nephrogenic diabetes insipidus defined by clinical symptoms, signs, biochemical findings, and for HR, radiological abnormalities; DDAVP = DDAVP (desmopressin) test – a normal response is defined by a urine osmolality ≥ 800 mOsm/kg.

**Table 2** Results of renal function investigations

| | Mean | Range | Normal range | No (%) of abnormal values |
|---|---|---|---|---|
| Glomerular | | | | |
| GFR (ml/min/1.73 $m^2$) | 93 | 44–189 | 90–175[#] | 61/123 (50%) |
| Serum creatinine (mmol/l) | 64 | 11–202 | ≤ 55–70* | 63/146 (47%) |
| Proximal tubular | | | | |
| Serum sodium (mmol/l) | 138 | 130–146 | 137–144 | 26/146 (18%) |
| Serum potassium (mmol/l) | 3.9 | 2.6–5.1 | 3.7–4.9 | 22/143 (15%) |
| Serum bicarbonate (mmol/l) | 21.9 | 8.5–30.0 | 18–26* | 22/95 (23%) |
| Serum phosphate (mmol/l) | 1.25 | 0.49–1.96 | 0.90–1.85* | 28/135 (21%) |
| Serum total calcium (mmol/l) | 2.38 | 1.74–2.75 | 2.30–2.63 | 12/139 (9%) |
| Serum magnesium (mmol/l) | 0.84 | 0.49–1.22 | 0.70–1.00*[α] | 6/134 (4%) |
| Serum urate (mmol/l) | 0.17 | 0.06–0.53 | 0.05–0.50* | 5/93 (5%) |
| FEsodium (%) | 0.9 | 0.02–3.7 | 0.2–1.9 | 2/103 (2%) |
| FEpotassium (%) | 18.6 | 1.4–68.2 | 3.5–30.6 | 8/100 (8%) |
| FEphosphate (%) | 20.7 | 1.5–117 | 2.2–20.2 | 19/103 (18%) |
| $Tm_p$/GFR (mmol/l) | 1.01 | –0.18–1.93 | 0.99–1.93*[β] | 45/103 (44%) |
| FEcalcium (%) | 2.4 | 0.07–33.3 | 0.1–5.6 | 0/95 |
| FEmagnesium (%) | 4.9 | 0.5–13.5 | 1.1–9.1 | 0/96 |
| FEglucose (%) | 13.5 | 0.01–250 | ≤ 0.05 | 52/59 (88%) |
| FEurate (%) | 2.9 | 0.1–7.1 | 7.0–12.0[δ] | 3/52 (6%) |
| Distal tubular | | | | |
| pH | 6.0 | 5.0–8.8 | ≤ 5.4** | 80/108 (74%) |
| Osmolality (mOsm/kg) | 674 | 121–1264 | ≥ 600** | 45/123 (37%) |
| General aspects | | | | |
| Urine albumin (mg/mmol creat) | 13.4 | 1.0–40.4 | < 10[λ] | 6/15 (40%) |
| Urine protein (mg/mmol creat) | 139.0 | 0.1–707.0 | < 20[π] | 3/67 (4%) |

*Normal range varies with age (Clayton et al, 1980; Brodehl et al, 1982; Skinner et al, 1991). **See text (Methods) (Skinner et al, 1991). Unless otherwise stated, normal ranges are derived from investigation of 105 healthy children aged 0.1–16.6 years (see text). Some published normal ranges were used, as listed below: # Barratt, (1974); α Clayton et al (1980); β Brodehl et al (1982); δ Grantham and Chonko (1991); λ Barratt et al (1970); π Elises et al (1988). Except for FEglucose, an elevated FE value is taken to indicate proximal tubular toxicity only if the serum concentration of the corresponding substance is below the normal range. A low FE value does not imply nephrotoxicity.

children and adolescents (aged 0.1–16.6 years, 27 male) attending the Royal Victoria Infirmary, Newcastle upon Tyne for investigation of a proven urinary tract infection (treated at least 1 month previously), in whom clinical examination, renal and urinary tract investigations and imaging proved to be normal (Table 2). Previously outlined normal ranges were used for age- or sex-dependent measures of toxicity, including biochemical variables (serum concentrations of creatinine, bicarbonate, phosphate and magnesium; and $Tm_p$/GFR) (Skinner et al, 1991), and systolic blood pressure (Children, 1987) (Table 2).

 *© 2000 Cancer Research Campaign*

### Statistical analysis

The normality or otherwise of variables was examined using the skewness and kurtosis tests. Multiple linear regression analysis was used to evaluate total ifosfamide dose, age at start of treatment, sex, ifosfamide schedule (short infusion or continuous infusion) and exposure to other potential nephrotoxins, namely aminoglycosides, vancomycin, acyclovir or amphotericin B (categorized as treatment at any time, or not, with each of these drugs) as predictors for nephrotoxicity measured by GFR, serum $PO_4$ and $HCO_3$ and $Tm_p$/GFR. Initially all predictor variables were included in multiple regression in a backward stepwise procedure and the least significant variables ($P > 0.2$) were rejected. Only total dose was found consistently to be significant (see Results); therefore univariate linear regression was performed with total dose only.

The nephrotoxicity score had a highly skewed distribution. Therefore, it was categorized as either none/mild (score 0–3) or moderate/severe (score ≥ 4), and stepwise logistic regression analysis was performed in which the 8 independent variables (dose, age, sex, schedule, and exposure to aminoglycosides, vancomycin, acyclovir or amphotericin B) were used to predict nephrotoxicity score. The above analyses were repeated after exclusion of 16 patients with other major risk factors for the development of nephrotoxicity (urinary tract obstruction at commencement of ifosfamide treatment, radiotherapy to renal tissue, cisplatin treatment). The absolute values of the individual elements of nephrotoxicity (e.g. GFR) in patients in whom the nephrotoxicity score could be calculated (i.e. patients with evaluable GFR, $Tm_p$/GFR, serum $HCO_3$ and EMUO) were compared with those in patients in whom the score could not be calculated, using unpaired *t*-tests for each variable. Analyses were performed using STATA statistical software (StataCorp, 1997).

### RESULTS

Those continuous measures of nephrotoxicity described below had an approximately normal distribution. Nephrotoxicity was observed in a substantial proportion of patients (Table 2), although considerable inter-individual variability was evident (Figures 1–2).

### Glomerular function

GFR was below 90 ml/min/1.73 $m^2$ in 61 of 123 evaluable patients (50%), and below 60 ml/min/1.73 $m^2$ (i.e. grade 2–4 toxicity) in 11 (9%). The $^{51}$Cr-EDTA plasma clearance technique was used in 58 of these and the $^{99m}$Tc-DTPA plasma clearance method in three. The serum creatinine concentration was elevated in 63 of 146 patients (43%). A statistically significant fall in GFR was observed in 67 patients in whom it was measured (using the same method) both at diagnosis and after completion of treatment (mean [95% confidence limits] fall 35.1 [22.1–47.9] ml/min/1.73 $m^2$; paired *t*-test, $t = 8.96$, $P < 0.001$).

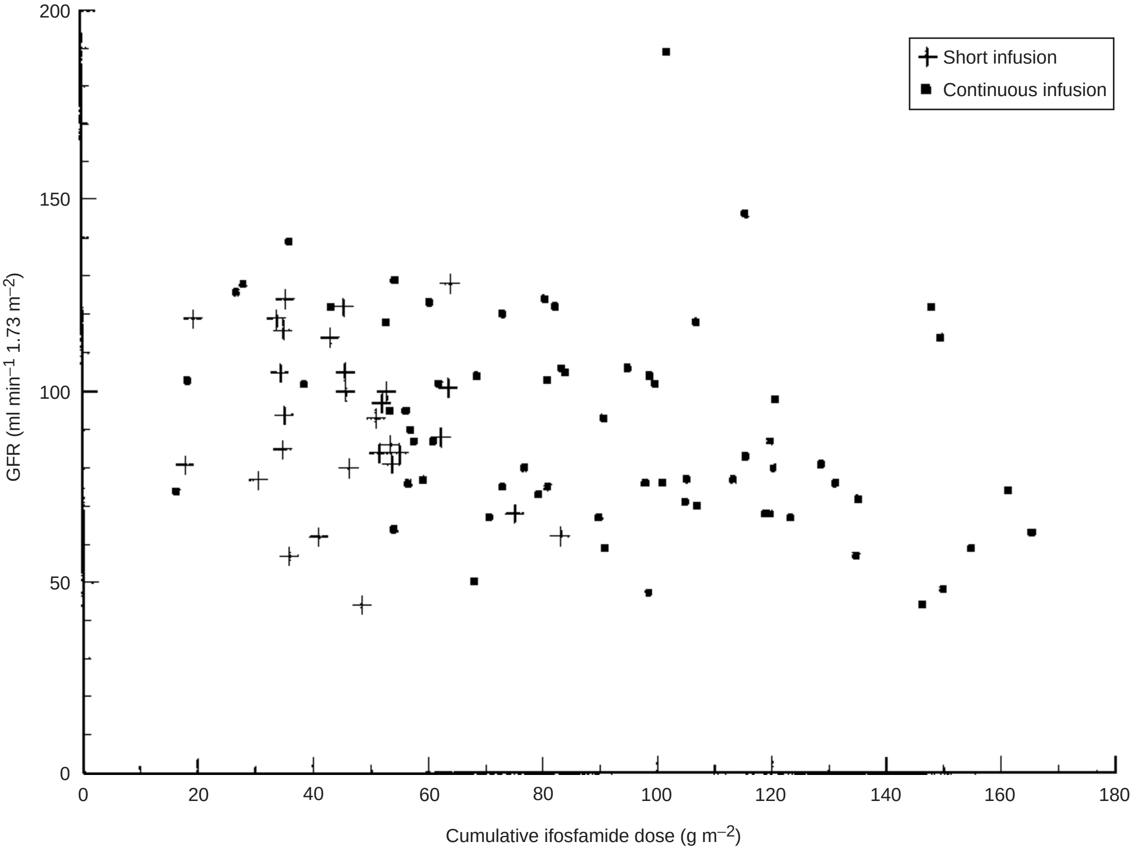

**Figure 1** Scatter plot showing relation between cumulative dose of ifosfamide received and GFR. Patients receiving ifosfamide as a short (3-h) infusion (+) or as a continuous infusion (■) are distinguished

© 2000 Cancer Research Campaign

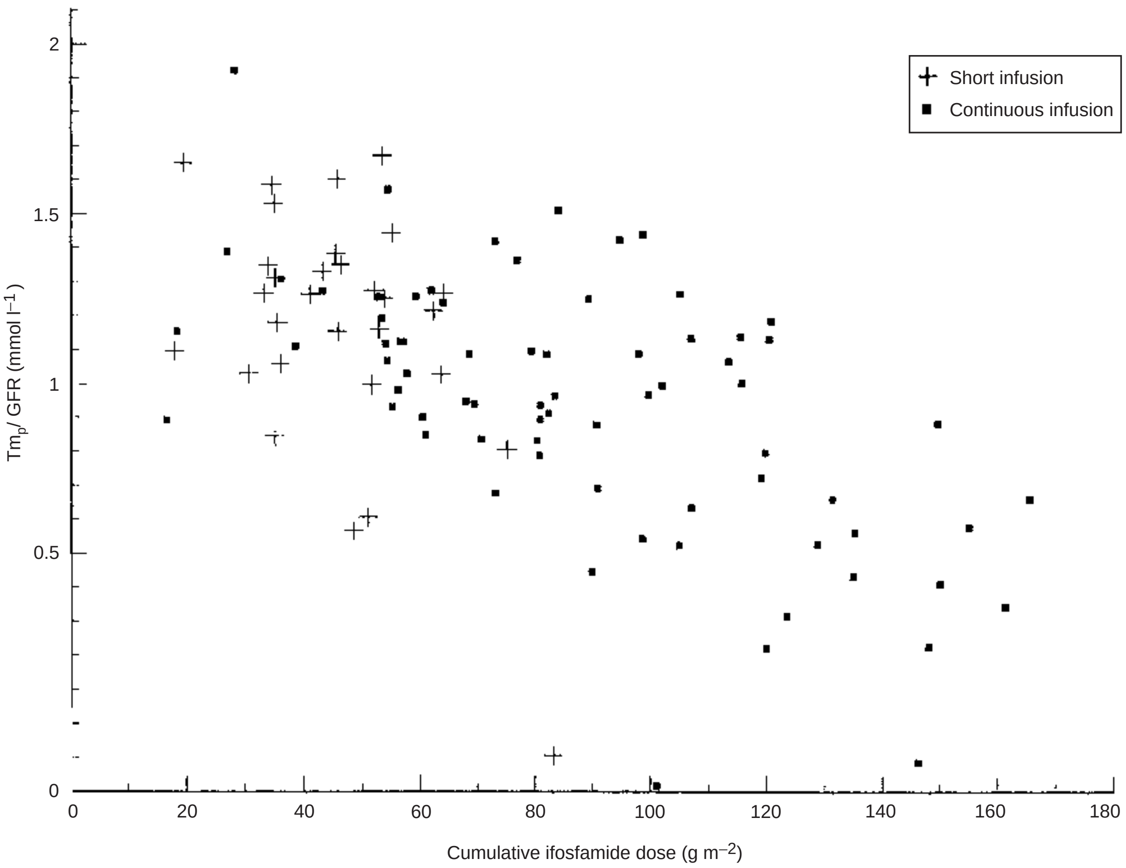

**Figure 2** Scatter plot showing relation between cumulative dose of ifosfamide received and $Tm_p$/GFR. Patients receiving ifosfamide as a short (3-h) infusion (+) or as a continuous infusion (■) are distinguished

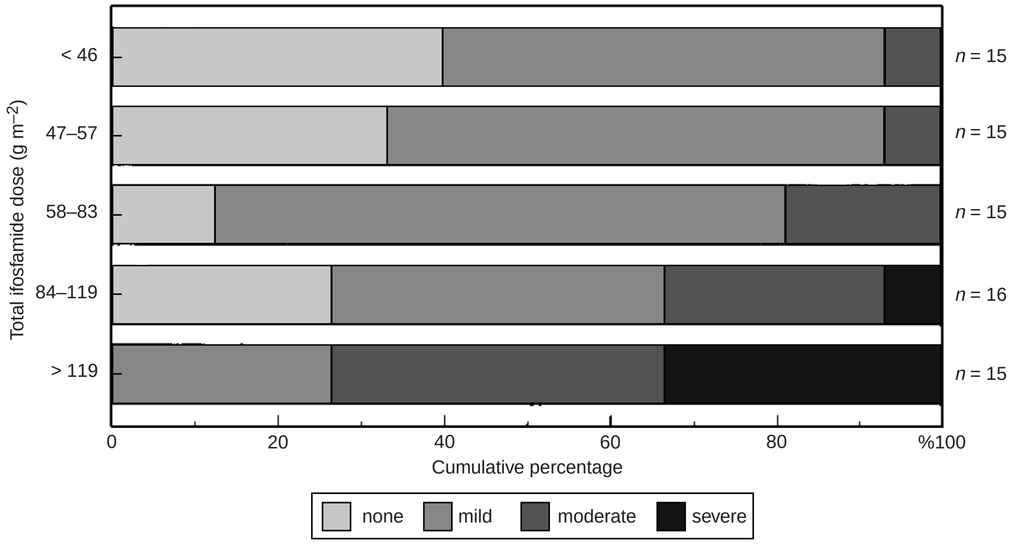

**Figure 3** The distribution of no, mild, moderate and severe nephrotoxicity amongst the 76 patients in whom the nephrotoxicity score was fully evaluable. The patients are divided into five groups according to the total dose of ifosfamide received

*© 2000 Cancer Research Campaign*

### Proximal tubular function

Hypophosphataemia was observed in 28 of 135 evaluable patients (21%), acidosis in 22 of 95 patients (23%), hypokalaemia in 22 of 143 (15%), hyponatraemia in 26 of 146 (18%), hypocalcaemia in 12 of 139 (9%), hypomagnesaemia in six of 134 (4%) and hypouricaemia in five of 93 (5%). Seven per cent of evaluable patients had grade 2–4 (moderate or severe) toxicity scores for serum $PO_4$, and 8% for serum $HCO_3$, but only 3% for serum potassium, 1% each for serum calcium and magnesium, and none for serum sodium.

Phosphaturia was demonstrated by a reduced $Tm_p$/GFR in 45 of 103 evaluable patients (44%), with grade 2–4 toxicity in 23 (22%). Only 19 of these 103 children (18%) had a high FEp. Excessive urinary excretion of other electrolytes was present in a smaller proportion of patients, with high fractional excretions of potassium in eight of 100 evaluable children, and of sodium in two of 103 (2%). The commonest urinary abnormality was glycosuria, shown by a high FEg in 52 of 59 evaluable patients (88%). Only three of 52 evaluable patients (6%) had a high fractional excretion of urate.

### Distal tubular function

The early morning urine was adequately acidified (pH ≤ 5.4) in only 28 of 108 evaluable patients (26%), and concentrated (EMUO ≥ 600 mOsm/kg) in 78 of 123 (63%).

### General aspects of renal function

Although only three of 67 evaluable patients (4%) had an elevated urine protein:creatinine concentration ratio, six of 15 (40%) had a high urine albumin:creatinine concentration ratio. Six of 127 evaluable patients (5%) had severe systolic hypertension; all other patients were normotensive.

The majority of patients with glomerular toxicity also had evidence of tubular impairment. Of 44 fully evaluable patients with GFR grade ≥ 1 (i.e. mild or greater toxicity), only 15 (34%) had grade 0 $Tm_p$/GFR and $HCO_3$. More strikingly, nearly all children with tubular toxicity had glomerular damage. Of 40 fully evaluable patients with grade ≥ 1 $Tm_p$/GFR and $HCO_3$, only one (3%) had grade 0 GFR.

### Nephrotoxicity grading

The nephrotoxicity score was fully evaluable (i.e. GFR, $Tm_p$/GFR, $HCO_3$ and EMUO all evaluable) in 76 patients. Of these, 17 (22%) had no, 38 (50%) mild, 15 (20%) moderate and six (8%) severe nephrotoxicity, using the definitions shown in Table 1. No significant difference was seen in the severity of nephrotoxicity between these patients and the other 72 in whom the nephrotoxicity score was not fully evaluable. No child had grade 4 toxicity for GFR, but ten of 103 (10%) had grade 4 toxicity for $Tm_p$/GFR, one of 95 (1%) for $HCO_3$, and five of 123 (4%) for EMUO.

### Risk factors

*GFR, serum $PO_4$, serum $HCO_3$ and $Tm_p$/GFR*

Multiple regression analysis revealed that only total dose exerted a consistent significant effect ($P < 0.05$) on GFR, serum $PO_4$ and $HCO_3$ and $Tm_p$/GFR. Univariate linear regression analysis revealed statistically significant and clinically important correlations between total ifosfamide dose and GFR ($P = 0.006$) (Figure 1), serum $PO_4$ ($P < 0.001$) and $HCO_3$ ($P < 0.001$), and $Tm_p$/GFR ($P < 0.001$) (Figure 2) (Table 3). Higher doses were associated with

**Table 3** Univariate and multivariate regression analysis of severity of nephrotoxicity on potential risk factors

| Measure of nephrotoxicity (*n* = number of evaluable patients) | Univariate | Multivariate | | | | | | | |
|---|---|---|---|---|---|---|---|---|---|
| | Change associated with increase in ifosfamide dose of 50 g m$^{-2}$ | Ifosfamide dose (g m$^{-2}$) | Age at treatment (years) | Sex | Ifosfamide schedule (short vs continuous infusion) | Exposure to amino-glycosides (yes vs no) | Exposure to vanco-mycin (yes vs no) | Exposure to acyclovir (yes vs no) | Exposure to ampho-tericin B (yes vs no) |
| GFR (*n* = 123) (ml/min/1.73 m$^2$) | −9.18* (−15.64 to −2.72) | −0.25* (−0.42 to −0.09) | – | – | −8.92 (−22.2 to 4.4) | – | – | – | – |
| Serum $PO_4$ (*n* = 135) (mmol/l) | −0.21* (−0.27 to −0.14) | −0.005* (−0.006 to −0.003) | −0.007 (−0.016 to 0.002) | −0.077 (−0.17 to −0.018) | – | – | – | – | −0.126* (– 0.002 to – 0.25) |
| Serum $HCO_3$ (*n* = 95) (mmol/l) | −1.93* (−2.87 to 0.99) | −0.04* (−0.06 to −0.02) | 0.28* (0.15 to 0.41) | – | – | 1.06 (−0.41 to 2.53) | – | – | −1.78 (−3.57 to 0.003) |
| $Tm_p$/GFR (*n* = 103) (mmol/l) | −0.32* (−0.41 to 0.24) | −0.006* (−0.008 to −0.005) | – | – | – | – | – | −0.16* (−0.30 to −0.03) | – |

Second column shows univariate linear regression coefficients, and third to tenth columns multiple regression coefficients, all expressed as mean (95% confidence limits). – denotes rejected from stepwise regression ($P > 0.2$); * $P < 0.05$. Reference ranges and abbreviations: GFR (glomerular filtration rate) = 90–175 ml/min/1.73 m$^2$; Serum $PO_4$ (phosphate) concentration = 0.90–1.85 mmol/l (age-related); Serum $HCO_3$ (bicarbonate) concentration = 18.0–26.0 mmol/l (age-related); Renal tubular threshold for phosphate ($Tm_p$/GFR) = 0.99–1.93 mmol/l (age-related).

**Table 4** Total ifosfamide dose as a risk factor for the development of proximal tubular toxicity

| | Total ifosfamide dose | | |
|---|---|---|---|
| | ≤ 79.9 (g/m²) | ≥ 80.0 (g/m²) | *P* |
| Serum $PO_4$ | 1.33 (0.03) | 1.10 (0.04) | < 0.001 |
| $Tm_p$/GFR | 1.18 (0.03) | 0.77 (0.06) | < 0.001 |
| Serum $HCO_3$ | 23.1 (0.4) | 20.3 (0.7) | < 0.001 |
| FEg | 1.8 (0.7) | 23.6 (8.8) | 0.02 |

Results expressed as mean (standard error). *P*-values relate to unpaired *t*-tests between the two different dose groups.

greater toxicity. However, due to considerable inter-patient variability, no entirely safe dose limit was discernible, but children receiving ≥ 80 g/m² suffered greater proximal tubular damage (Figure 2, Table 4). The other predictor variables studied in the multivariate analysis had no independent effect, with the exceptions of relationships between younger age at treatment and lower serum $HCO_3$ ($P < 0.001$), acyclovir exposure and lower $Tm_p$/GFR ($P = 0.02$), and amphotericin B exposure and lower serum $PO_4$ ($P = 0.049$) (Table 3).

### *Nephrotoxicity score*

Multiple logistic regression revealed that only total ifosfamide dose had a significant predictive influence ($P = 0.001$) on nephrotoxicity score (none/mild vs moderate/severe); other potential risk factors had no significant effect ($P > 0.1$ in all cases). An increase in ifosfamide dose of 50 g/m² increased the risk of moderate/severe (compared to no/mild) nephrotoxicity by an odds ratio of 6.8 (95% confidence limits 2.7–16.9). The importance of ifosfamide dose in determining the likelihood of no, mild, moderate or severe nephrotoxicity is illustrated in Figure 3.

Exclusion of the 16 patients with other major risk factors for the development of nephrotoxicity did not change the outcome of these analyses – total dose remained the only consistently significant predictor of nephrotoxicity.

### DISCUSSION

Although early studies failed to reveal any evidence of nephrotoxicity in children receiving ifosfamide (de Kraker and Voute, 1984; Gasparini, 1986; Biron et al, 1987; Kellie et al, 1988; Demeocq et al, 1989), subsequent reports described a characteristic pattern of proximal renal tubular damage, often accompanied by glomerular and sometimes by distal tubular impairment (Smeitink et al, 1988; Burk et al, 1990; Skinner et al, 1990; Pratt et al, 1991; Suarez et al, 1991; Caron et al, 1992; Shore et al, 1992; De Schepper et al, 1993; Arndt et al, 1994; Ashraf et al, 1994). Such toxicity persisted long after discontinuation of ifosfamide treatment in many patients, often presenting with clinical manifestations due to the Fanconi syndrome (Skinner et al, 1993). It is now clear that there is much inter-individual variability in the onset, nature and severity of renal toxicity due to ifosfamide. Many children suffer little or no renal toxicity, but a few are severely affected (Smeitink et al, 1988; Skinner et al, 1990, 1993). Acute tubular toxicity may follow the first course of treatment (Heney et al, 1989; Devalck et al, 1991), or chronic glomerular and tubular damage may present many months after completion of ifosfamide (Moncrieff and Foot, 1989; De Schepper et al, 1991; Suarez et al, 1991; Caron et al, 1992).

The reported incidence of severe chronic nephrotoxicity in children has varied widely from 1.4% (Pratt et al, 1991) to about 30% (De Schepper et al, 1993), probably depending on the distribution of risk factors amongst different patient groups and on the sensitivity of the methods used to detect renal damage. The commonest clinical sequelae of ifosfamide nephrotoxicity include hypophosphatemia, which may lead to rickets (Moncrieff and Foot, 1989; Burk et al, 1990; Skinner et al, 1990; De Schepper et al, 1991; Pratt et al, 1991; Suarez et al, 1991; De Schepper et al, 1993), and renal tubular acidosis (Heney et al, 1989; Suarez et al, 1991), both of which may impair growth (De Schepper et al, 1991). Nephrogenic diabetes insipidus may occur (Smeitink et al, 1988; Skinner et al, 1990).

The degree of reversibility of ifosfamide nephrotoxicity remains uncertain. Although partial improvement may occur, including resolution of rickets without specific treatment (Van Gool et al, 1992) and improvement in biochemical abnormalities in some children (Suarez et al, 1991; Caron et al, 1992), there is only one published description of complete recovery from severe, chronic glomerular and tubular impairment in children (Ashraf et al, 1997). Indeed, there is evidence that glomerular (Burk et al, 1990; Prasad et al, 1996) and tubular (Caron et al, 1992) toxicity may progress over a period of months or years following completion of ifosfamide treatment.

Several risk factors for the development of nephrotoxicity after ifosfamide have been suggested, including age, the total dose of ifosfamide received, the method of drug administration, previous or concurrent treatment with cisplatin, prior nephrectomy and the presence of pre-existing renal impairment or infiltration by tumour (Skinner et al, 1993). Furthermore, quantitative inter-patient variability in ifosfamide metabolism may determine individual risk (Skinner et al, 1993).

Although ifosfamide nephrotoxicity may occur at any age (Skinner et al, 1993), most published reports of severe toxicity have been in young children, who may be more susceptible to proximal tubular toxicity, (Suarez et al, 1991; Caron et al, 1992; Shore et al, 1992; De Schepper et al, 1993; Skinner et al, 1993; Raney et al, 1994), due to a combination of anatomical, biochemical and physiological factors (Fetterman et al, 1965). Although extensive renal damage has occurred after total ifosfamide doses of between 12 and 60 g/m² (Smeitink et al, 1988; Heney et al, 1989; Moncrieff and Foot, 1989; Burk et al, 1990; Devalck et al, 1991), several authors have suggested that children receiving higher cumulative doses (> 60 or > 72 g/m²) have a greater risk of nephrotoxicity (Bisogno et al, 1993; De Schepper et al, 1993; Skinner et al, 1993; Raney et al, 1994).

Despite documented variability in ifosfamide metabolism with different schedules, no published evidence is yet available which shows convincingly that any one administration regiment of ifosfamide and mesna is superior in terms of improved efficacy or reduced toxicity (Skinner et al, 1993). Some studies have found an increased incidence of severe ifosfamide nephrotoxicity in patients who have also received cisplatin (Moncrieff and Foot, 1989; Pratt et al, 1991; Shore et al, 1992; Rossi et al, 1994) or who had previously undergone unilateral nephrectomy (Burk et al, 1990; Rossi et al, 1994). Furthermore, there are several case reports of severe renal damage after ifosfamide treatment in patients with renal infiltration by tumour (Bremner et al, 1974; Holoye et al, 1982), or those with pre-existing renal impairment (Wheeler et al, 1986; Davies et al, 1989), but it is not known whether the overall incidence of nephrotoxicity is increased in these patients.

 *© 2000 Cancer Research Campaign*

There is still much uncertainty about role of each of these risk factors, especially age, dose and cisplatin treatment, but ascertainment of the most important might enable prediction and careful monitoring of 'high risk' patients or. A major aim of this study was therefore to clarify which of the above risk factors was most important in a large group of children and adolescents receiving ifosfamide in standard treatment protocols in the UK.

The commonest abnormal finding was of proximal tubular toxicity in association with glomerular impairment. Fewer patients had isolated glomerular, and very few isolated tubular, damage. Additional biochemical abnormalities most likely to be due to proximal tubular toxicity were seen in a minority of patients, but the magnitude of these latter abnormalities was considerably less than that of the hypophosphataemia and acidosis.

The finding that GFR was reduced in 50% of patients demonstrates that glomerular impairment is very common when accurate measurements (e.g. radioisotopic plasma clearance methods) are used. Although reports of chronic renal failure are very rare (Sangster et al, 1984), this frequency of subclinical glomerular damage leads to concern about the future prognosis for renal function in some of these patients. The glomerulotoxic nature of ifosfamide was shown clearly by the mean fall of 35 ml/min/1.73 $m^2$ in the 66 patients in whom GFR was measured before and after chemotherapy.

The system of nephrotoxicity grading (Skinner et al, 1993) employed in this study provided an *objective* overall measure of the severity of *clinically relevant* nephrotoxicity, in contrast to the more subjective score proposed previously by Caron et al (1992). The demonstration that only 22% of 76 fully evaluable patients had no nephrotoxicity, and that 28% had moderate or severe clinically relevant toxicity, is therefore particularly worrying. It might be argued that patients with severe toxicity were more likely to be entered into this study, and to be evaluated fully. However, this is unlikely to have been the case since no selection bias within centres was apparent with respect to patient entry. In total, 91% of the 119 potentially eligible patients identified at the start of the study were either studied or excluded due to death or progressive disease. Furthermore, there was no significant difference in the severity of proximal tubular toxicity between the 76 patients with fully evaluable nephrotoxicity scores and the 72 without.

The analysis of risk factors for the development of ifosfamide nephrotoxicity in this population of patients, none of whom had undergone nephrectomy and only three of whom had received cisplatin, has demonstrated clearly that total dose is the most important risk factor. This is of great practical importance because in many countries ifosfamide is seldom used in combination with cisplatin or in patients who have undergone unilateral nephrectomy. Therefore total ifosfamide dose is probably more generally relevant as a major risk factor than prior or concurrent cisplatin treatment and nephrectomy. Although it is clear from Figures 1 and 2 that the considerable inter-patient variability in the severity of nephrotoxicity, even between patients receiving similar doses, prevents definition of an entirely 'safe' dose limit, Figure 3 demonstrates that no patients receiving < 84 g/$m^2$ suffered severe, and only 20% moderate, nephrotoxicity; whilst of those receiving > 119 g/$m^2$, 33% suffered severe and 40% moderate nephrotoxicity. Clearly, clinically significant nephrotoxicity is relatively infrequent below 84 g/$m^2$ but unacceptably common above 119 g/$m^2$.

Another important finding is that age is not a major independent risk factor. The relevance of the statistically significant relationship between younger age and lower serum $HCO_3$ is uncertain since healthy infants have lower serum $HCO_3$ concentrations (Skinner et al, 1991). However, caution is still necessary in younger patients, in whom the consequences of nephrotoxicity, including growth impairment, may be greater.

This is the largest published study of ifosfamide nephrotoxicity in which investigations have been performed prospectively. However, a few potential criticisms of the study may be made. In particular, the multi-centre design might have led to bias in patient selection. However, there was no evidence of such bias, and a consistent investigatory protocol was used in all centres, incorporating well established and straightforward clinical investigations. Some of these investigations have normal ranges that vary with age, but this was accounted for in the analysis of the frequency of toxicity. It is theoretically possible that the higher normal values of serum $PO_4$ and of $Tm_p$/GFR in younger children may have obscured the importance of younger age as a risk factor for this aspect of proximal tubular toxicity. However, there was no other suggestion that age was a significant predictor of nephrotoxicity, even with measures of renal function that do not vary significantly over the age range studied (e.g. GFR, which is stable after the age of 2 years).

Although ifosfamide administration schedule was included as a predictor in the multiple regression model, this analysis may not have been able to distinguish any potential independent effect of schedule from that of the total ifosfamide dose received in view of the close relationship between the schedule used and total dose received. However, inspection of Figures 1 and 2 does not suggest that schedule has any important independent influence. The majority of patients studied had been exposed to other potential nephrotoxic insults. Exclusion of 16 patients with the most important potential risk factors (urinary tract obstruction, renal radiotherapy, cisplatin) did not alter the results, and analysis of the possible importance of exposure to other nephrotoxic drugs (aminoglycosides, vancomycin, acyclovir, amphotericin B) did not suggest any consistent pattern of increased nephrotoxicity. However, exposure to these drugs was only recorded as present or absent, so interpretation of their possible additive effects in ifosfamide nephrotoxicity is limited.

The cross-sectional nature of the study is unlikely to have biased the results significantly since there is no published evidence of consistent important changes in the severity of chronic ifosfamide nephrotoxicity with time after completion of treatment, and any such changes would have been highly unlikely to produce systematically a spurious dose effect of the magnitude observed in this study. In any case the occurrence of chronic nephrotoxicity at any time after completion of treatment is of concern.

The results of this study suggest that future strategies to prevent ifosfamide nephrotoxicity in children and adolescents should be centred around dose limitation, but also imply that this approach, whilst reducing the frequency of clinically significant nephrotoxicity, will not prevent all cases. It is possible that some of the inter-patient variability in the severity of toxicity reflects corresponding differences between individuals in the pharmacokinetics and metabolism of ifosfamide, but this remains unproven (Boddy et al, 1996). Identification of such a relationship and its use to enable prediction of the risk of subsequent nephrotoxicity clearly would be of great value. An alternative approach of using the occurrence

© 2000 Cancer Research Campaign 

and magnitude of early subclinical nephrotoxicity to enable prediction of subsequent chronic toxicity is under investigation (Skinner et al, 1994). Although it appears unlikely that different ifosfamide schedules are associated with different risks of toxicity, this issue should be addressed in a comparative study that includes endpoints of efficacy as well as toxicity.

In conclusion, this large and comprehensive prospective study has shown that moderate and severe nephrotoxicity are common after ifosfamide treatment in children and adolescents, affecting 28% of patients. Multivariate analysis revealed that total ifosfamide dose was the only significant risk factor for the development of toxicity in this group of patients, only three of whom received cisplatin. Although it was not possible to specify a clinically realistic total dose below which toxicity was never observed, use of doses below 84 g/m$^2$ will reduce the frequency of clinically significant nephrotoxicity.

### ACKNOWLEDGEMENTS

We wish to thank the paediatric oncologists, research nurses and data managers at the 11 UKCCSG centres (listed below) that participated in this study, and Mr John Imeson for his assistance with the study's design. The following centres contributed patients – Birmingham, Cambridge, Cardiff, Dublin, Edinburgh, Leeds, Leicester, Newcastle, Royal Marsden Hospital, Sheffield and Southampton. The UKCCSG is supported by the Cancer Research Campaign. Dr Skinner and Mr Cotterill received financial support from the North of England Children's Cancer Research Fund.

### REFERENCES

Arndt C, Morgenstern B, Wilson D, Liedtke R and Miser J (1994) Renal function in children and adolescents following 72 g/m$^2$ of ifosfamide. *Cancer Chemother Pharmacol* **34**: 431–433

Ashraf MS, Brady J, Breatnach F, Deasy PF and O'Meara A (1994) Ifosfamide nephrotoxicity in paediatric cancer patients. *Eur J Paediatr* **153**: 90–94

Ashraf MS, Skinner R, English MW, Craft AW and Pearson ADJ (1997) Late reversibility of chronic ifosfamide-associated nephrotoxicity in a child. *Med Pediatr Oncol* **28**: 62–64

Barratt TM (1974) Assessment of renal function in children. In: *Modern Trends in Paediatrics, 4*. Apley J (ed), pp. 181–215. Butterworth, London

Barratt TM, McLaine PN and Soothill JF (1970) Albumin excretion as a measure of glomerular dysfunction in children. *Arch Dis Child* **45**: 496–501

Biron P, Philip T, Pinkerton R, Provensal C and Brunat-Mentigny M (1987) A regimen with ifosfamide and cis-platinum in osteosarcomas: a phase-II study. *Contrib Oncol* **26**: 125–130

Bisogno G, Dussini N, Moggia D, Perilongo G and Carli M (1993) Pattern of aminoaciduria in patients with soft-tissue sarcomas treated with ifosfamide-containing regimens. *Am J Pediatr Hematol Oncol* **15**: S5–S9

Bleyer WA (1990) The impact of childhood cancer on the United States and the world. *Ca: Cancer J Clin* **40**: 355–367

Boddy AV, English MW, Pearson ADJ, Idle JR and Skinner R (1996) Ifosfamide nephrotoxicity: limited influence of metabolism and mode of administration during repeated therapy in paediatrics. *Eur J Cancer* **32A**: 1179–1184

Bremner DN, McCormick JS and Thomson JWW (1974) Clinical trial of isophosphamide (NSC-109724) – results and side effects. *Cancer Chemother Rep* **58**: 889–893

Brodehl J, Gellison K and Weber HP (1982) Postnatal development of tubular phosphate reabsorption. *Clin Nephrol* **17**: 163–171

Burk CD, Restaino I, Kaplan BS and Meadows AT (1990) Ifosfamide-induced renal tubular dysfunction and rickets in children with Wilms tumor. *J Pediatr* **117**: 331–335

Caron HN, Abeling N, van Gennip A, de Kraker J and Voute PA (1992) Hyperaminoaciduria identifies patients at risk of developing renal tubular toxicity associated with ifosfamide and platinate containing regimens. *Med Pediatr Oncol* **20**: 42–47

Clayton BE, Jenkins P and Round JM (1980) *Paediatric Chemical Pathology.* Blackwell Scientific, Oxford

Craft A, Cotterill S, Malcolm A, Spooner D, Grimer R, Souhami R, Imeson J and Lewis I (1998) Ifosfamide-containing chemotherapy in Ewing's sarcoma: The Second United Kingdom Children's Cancer Study Group and the Medical Research Council Ewing's Tumor Study. *J Clin Oncol* **16**: 3628–3633

Davies SM, Pearson ADJ and Craft AW (1989) Toxicity of high-dose ifosfamide in children. *Cancer Chemother Pharmacol* **24**: S8–S10

de Kraker J and Voute PA (1984) Ifosfamide and vincristine in paediatric tumours. A phase II study. *Eur Paediatr Haematol Oncol* **1**: 47–50

De Schepper J, Stevens G, Verboven M, Baeta C and Otten J (1991) Ifosfamide-induced Fanconi's syndrome with growth failure in a 2-year-old child. *Am J Pediatr Hematol Oncol* **13**: 39–41

De Schepper J, Hachimi-Idrissi S, Verboven M, Piepsz A and Otten J (1993) Renal function abnormalities after ifosfamide treatment in children. *Acta Paediatr* **82**: 373–376

Demeocq F, Oberlin O, Benz-Lemoine E, Biolletot A, Gentet JC, Zucker JM, Behar C, Poutard P, Olive D, Brunat-Mentigny M, Demaille MC, Patte C, Contesso G and Lemerle J (1989) Initial chemotherapy including ifosfamide in the management of Ewing's sarcoma: preliminary results. A protocol of the French Pediatric Oncology Society (SFOP). *Cancer Chemother Pharmacol* **24**: S45–S47

Devalck C, Ismaili K, Ferster A and Saribou E (1991) Acute ifosfamide-induced proximal tubular toxic reaction (letter). *J Pediatr* **118**: 325–326

Elises JS, Griffiths PD, Hocking MD, Taylor CM and White RHR (1988) Simplified quantification of urinary protein excretion in children. *Clin Nephrol* **30**: 225–229

Fetterman GH, Shuplock NA, Philipp FJ and Gregg HS (1965) The growth and maturation of human glomeruli and proximal convolutions from term to adulthood. *Pediatr* **35**: 601–619

Gasparini M (1986) High dose ifosfamide alone and in combination for solid malignancies in childhood. *Cancer Chemother Pharmacol* **18**: S18

Grantham JJ and Chonko AM (1991) Renal handling of organic anions and cations; excretion of uric acid. In: *The Kidney*, Brenner BM and Rector FC Jr (eds), Vol. I, pp. 483–509. WB Saunders, Philadelphia

Hawkins MM and Stevens MCG (1996) The long-term survivors. *Br Med Bull* **52**: 898–923

Heney D, Lewis IJ and Bailey CC (1989) Acute ifosfamide-induced tubular toxicity (letter). *Lancet* **ii**: 103–104

Holoye PY, Anderson T, Duelge J, Hansen RM and Ritch PS (1982) Use and safety of high-dose ifosfamide. *Semin Oncol* **9**: 78–86

Kellie SJ, de Kraker J, Lilleyman JS, Bowman A and Pritchard J (1988) Ifosfamide in previously untreated disseminated neuroblastoma. *Eur J Cancer Clin Oncol* **24**: 903–908

Moncrieff M and Foot A (1989) Fanconi syndrome after ifosfamide. *Cancer Chemother Pharmacol* **23**: 121–122

Prasad VK, Lewis IJ, Aparicio SR, Heney D, Hale JP, Bailey CC and Kinsey SE (1996) Progressive glomerular toxicity of ifosfamide in children. *Med Pediatr Oncol* **27**: 149–155

Pratt CB, Douglass EC, Etcubanas EL, Goren MP, Green AA, Hayes FA, Horowitz ME, Meyer WH, Thompson EI and Wilimas JA (1989) Ifosfamide in pediatric malignant solid tumors. *Cancer Chemother Pharmacol* **24**: S24–S27

Pratt CB, Meyer WH, Jenkins JJ, Avery L, McKay CP, Wyatt RJ and Hancock ML (1991) Ifosfamide, Fanconi's syndrome, and rickets. *J Clin Oncol* **9**: 1495–1499

Raney B, Ensign LG, Foreman J, Khan F, Newton W, Ortega J, Ragab A, Wharam M, Wiener E and Maurer H (1994) Renal toxicity of ifosfamide in pilot regimens of the Intergroup Rhabdomyosarcoma Study for patients with gross residual disease. *Am J Pediatr Hematol Oncol* **16**: 286–295

Rossi R, Godde A, Kleinebrand A, Riepenhausen M, Boos J, Ritter J and Jurgens H (1994) Unilateral nephrectomy and cisplatin as risk factors of ifosfamide-induced nephrotoxicity: analysis of 120 patients. *J Clin Oncol* **12**: 159–165

Sangster G, Kaye SB, Calman KC and Dalton JF (1984) Failure of 2-mercaptoethane sulphonate sodium (mesna) to protect against ifosfamide nephrotoxicity. *Eur J Cancer Clin Oncol* **20**: 435–436

Shaw PJ and Eden OB (1990) Ifosfamide in paediatric oncology: tried but not tested? *Lancet* **i**: 1022–1023

Shore R, Greenberg M, Geary D and Koren G (1992) Iphosphamide-induced nephrotoxicity in children. *Pediatr Nephrol* **6**: 162–165

Skinner R, Pearson ADJ, Price L, Coulthard MG and Craft AW (1990) Nephrotoxicity after ifosfamide. *Arch Dis Child* **65**: 732–738

Skinner R, Pearson ADJ, Coulthard MG, Skillen AW, Hodson AW, Goldfinch ME, Gibb I and Craft AW (1991) Assessment of chemotherapy-associated nephrotoxicity in children with cancer. *Cancer Chemother Pharmacol* **28**: 81–92

 © 2000 Cancer Research Campaign

Skinner R, Sharkey IM, Pearson ADJ and Craft AW (1993) Ifosfamide, mesna and nephrotoxicity in children. *J Clin Oncol* **11**: 173–190

Skinner R, Pearson ADJ, Price L, Wyliie RA, English MW, Hodson AW, Skillen AW, Coulthard MG and Craft AW (1994) Ifosfamide nephrotoxicity in children: early detection and prediction of severity (abstract). *Med Pediatr Oncol* **23**: 178

Skinner R, Pearson ADJ, English MW, Price L, Wyllie RA, Coulthard MG and Craft AW (1996) Chronic ifosfamide nephrotoxicity in children. *Lancet* **348**: 578–580

Smeitink J, Verreussel M, Schroder C and Lippens R (1988) Nephrotoxicity associated with ifosfamide. *Eur J Pediatr* **148**: 164–166

StataCorp (1997) *Stata Statistical Software: Release 5.0*. Stata Corporation College Station, TX

Suarez A, McDowell H, Niaudet P, Comay E and Flamant F (1991) Long-term follow-up of ifosfamide renal toxicity in children treated for malignant mesenchymal tumors: an International Society of Pediatric Oncology report. *J Clin Oncol* **9**: 2177–2182

Task Force on Blood Pressure Control in Children (1987) Report of the Second Task Force on Blood Pressure Control in Children – 1987. *Pediatrics* **79**: 1–25

Van Gool S, Brock P, Wijndaele G, Van de Casseye W, Kruger M, Proesmans W and Casteels-Van Daele M (1992) Reversible hypophosphatemic rickets following ifosfamide treatment. *Med Pediatr Oncol* **20**: 254–257

Wheeler BM, Loehrer PJ, Williams SD and Einhorn LH (1986) Ifosfamide in refractory male germ cell tumours. *J Clin Oncol* **4**: 28–34

Zalupski M and Baker LH (1988) Ifosfamide. *J Natl Cancer Inst* **80**: 556–566

*© 2000 Cancer Research Campaign*
